# Eye care delivery models to improve access to eye care for Indigenous peoples in high-income countries: a scoping review

**DOI:** 10.1136/bmjgh-2020-004484

**Published:** 2021-03-24

**Authors:** Helen Burn, Lisa Hamm, Joanna Black, Anthea Burnett, Matire Harwood, Matthew J Burton, Jennifer R Evans, Jacqueline Ramke

**Affiliations:** 1 International Centre for Eye Health, London School of Hygiene & Tropical Medicine, London, UK; 2 School of Optometry and Vision Science, Faculty of Medicine and Health Sciences, The University of Auckland, Auckland, New Zealand; 3 School of Optometry and Vision Science, University of New South Wales, Sydney, New South Wales, Australia; 4 General Practice and Primary Healthcare, Faculty of Medicine and Health Sciences, University of Auckland, Auckland, New Zealand; 5 Moorfields Eye Hospital NHS Foundation Trust, London, UK

**Keywords:** eye diseases, systematic review, health systems

## Abstract

**Purpose:**

Globally, there are ~370 million Indigenous peoples. Indigenous peoples typically experience worse health compared with non-Indigenous people, including higher rates of avoidable vision impairment. Much of this gap in eye health can be attributed to barriers that impede access to eye care services. We conducted a scoping review to identify and summarise service delivery models designed to improve access to eye care for Indigenous peoples in high-income countries.

**Methods:**

Searches were conducted on MEDLINE, Embase and Global Health in January 2019 and updated in July 2020. All study designs were eligible if they described a model of eye care service delivery aimed at populations with over 50% Indigenous peoples. Two reviewers independently screened titles, abstracts and full-text articles and completed data charting. We extracted data on publication details, study context, service delivery interventions, outcomes and evaluations, engagement with Indigenous peoples and access dimensions targeted. We summarised findings descriptively following thematic analysis.

**Results:**

We screened 2604 abstracts and 67 studies fulfilled our eligibility criteria. Studies were focused on Indigenous peoples in Australia (n=45), USA (n=11), Canada (n=7), New Zealand (n=2), Taiwan (n=1) and Greenland (n=1). The main disease focus was diabetic retinopathy (n=30, 45%), followed by ‘all eye care’ (n=16, 24%). Most studies focused on targeted interventions to increase availability of services. Fewer than one-third of studies reported involving Indigenous communities when designing the service. 41 studies reflected on whether the model improved access, but none undertook rigorous evaluation or quantitative assessment.

**Conclusions:**

The geographical and clinical scope of service delivery models to improve access to eye care for Indigenous peoples in high-income countries is narrow, with most studies focused on Australia and services for diabetic retinopathy. More and better engagement with Indigenous communities is required to design and implement accessible eye care services.

**Key questions**

**What is already known?**

- Indigenous peoples globally experience worse health outcomes compared with non-Indigenous people, including a higher prevalence of avoidable blindness and vision impairment, most commonly due to cataract and refractive error.
- In high-income countries, much of this inequality is a result of lack of access to appropriate eye care services for Indigenous peoples.

**What are the new findings?**

- There is a narrow range of evidence on how to deliver accessible eye care services for Indigenous peoples in high-income countries.
- The majority of the reports identified in our review were conducted in Australia and focused on teleophthalmology screening for diabetic retinopathy.
- Less than half of all reports described service delivery models that aimed to be culturally appropriate or engage Indigenous communities during service design and implementation.
- The methodological approaches used to describe and evaluate interventions to improve access to eye care could be strengthened to provide more robust evidence on effectiveness.

**What do the new findings imply?**

- Future research needs a greater geographical scope and should include services to address the leading causes of vision loss in Indigenous peoples: cataract and refractive error.
- The design and evaluation of eye care service delivery models would benefit from consideration of all five dimensions of access (*approachability*, *acceptability*, *availability*, *affordability* and *appropriateness*) and more partnership with Indigenous peoples.

## Introduction

Indigenous peoples are custodians, guardians and practitioners of unique ways of life.^1^ Indigenous peoples have displayed strength and persistence in preserving and continuing their culture despite a shared history of violent colonisation and violation of human rights. In 2015, most countries signed up to achieve the Sustainable Development Goals (SDGs) by 2030, with an overarching aim to leave no one behind.^2^ In the 90 countries where Indigenous peoples live, of whom there are an estimated 370 million, they are among the most marginalised and should therefore be a priority group in the SDG era.^3^ The term Indigenous people is used to describe many diverse peoples and cultures. For this review we use the definition of Indigenous peoples provided by the United Nations Permanent Forum on Indigenous Issues (UNPFII).^3 4^

Indigenous peoples across the globe have poorer health and social outcomes compared with non-Indigenous people, including dying younger, having higher rates of infant mortality and poverty, and lower educational attainment.^5^ The 2015 United Nations report, *State of the World’s Indigenous Peoples*, stated that ‘Indigenous peoples’ access to adequate health care remains one of the most challenging and complex areas’.^6^ While 80% of Indigenous peoples live in low-income and middle-income countries in Asia, Latin America and Africa, the report recognised that in high-income countries Indigenous peoples experience significant health disadvantage compared with non-Indigenous people as a result of institutionalised discrimination and marginalisation.^6 7^ For example, in 2012 the median life expectancy for Indigenous Australians was 10 years lower than for non-Indigenous Australians.^8^

One area of concern for Indigenous peoples is eye health. Vision impairment surveys tend not to include subanalysis by Indigeneity, so in many countries the prevalence of vision impairment among Indigenous communities is unknown.^9^ Australia is the only high-income country to have carried out a nationwide survey of the prevalence and causes of vision loss comparing Indigenous and non-Indigenous Australians. The 2016 survey found that the prevalence of vision impairment was 2.8 times greater in Indigenous Australians (17.7%, 95% CI 14.5 to 21.0) compared with non-Indigenous Australians (6.4%, 95% CI 5.2 to 7.6) after adjusting for age and gender (p<0.001).^10^ Much of this disparity in vision impairment can be attributed to reduced access to eye care services, distrust of health services and a lack of cultural safety and non-clinical support systems.^11^ Indigenous participants had lower prevalence of eye care examinations compared with non-Indigenous participants, with geographical remoteness further reducing the likelihood of having had an eye examination in the past 2 years.^12 13^ Canada, the USA and New Zealand have had no nationwide survey of Indigenous eye health, but smaller studies reporting prevalence of vision impairment, blindness or specific ophthalmic conditions have consistently shown a higher burden among Indigenous compared with non-Indigenous communities.^14–20^ Again, access to eye care services is highlighted as a key factor in maintaining this population inequality, for example, eye services not being available in locations with majority Indigenous peoples, the prohibitive cost of travelling to and accessing clinical care, a lack of integrated culturally appropriate eye services and a lack of consistent skills in cultural safety for those delivering care.^12 21^

Strategies to improve access to eye care services for Indigenous peoples must be informed by evidence and include the perspectives of those people currently ‘missing out’. This scoping review aims to summarise the existing literature on service delivery models designed to improve access to eye care services for Indigenous peoples. This work fed into the *Lancet Global Health* Commission on Global Eye Health.^22^

This review is focused on high-income countries and aimed to answer the following questions:

- What is the quantity and the characteristics of published reports describing service delivery models to improve access to eye care for Indigenous peoples in high-income countries?
- What methods and interventions are used by these service delivery models to improve access to eye care?
- What are the current gaps in the literature and what lessons can be learnt regarding models that have been successful in improving access?

We defined *eye care service delivery models* as any organised programme designed to provide or improve eye care services, ranging from non-specialised primary healthcare to tertiary ophthalmic care.^23^ Our definition of *access* was guided by the conceptual framework of patient-centred healthcare access by Levesque *et al*,^24^ which emphasises the importance of both the supply and demand sides of healthcare access.

## Methods

### Protocol and registration

The protocol for this review has been previously published.^23^ We have reported this review following the Preferred Reporting Items for Systematic Reviews and Meta-Analyses for Scoping Reviews guideline.^25^

### Patient and public involvement

It was not feasible to include patient and public engagement in this research.

### Eligibility criteria

Our eligibility criteria were as follows:

- *Population:* the target population of the service delivery model was Indigenous peoples, as defined by the United Nations.^4^ If the target population was not exclusively Indigenous, we included studies where 50% or more of the population were Indigenous.
- *Intervention:* any service delivery model to improve access to eye care. These could include theoretical modelling of a service or a description and/or evaluation of an existing implemented service delivery model. If the report gave a clear description of the service delivery model within the methodology or discussion, it was included even if its primary aim was not to describe or evaluate a service delivery model. For example, reports with a primary aim of describing disease prevalence but which also describe an eye service delivery model within the report were included.
- *Setting:* high-income country (as defined by the World Bank in 2019).^26^
- *Comparator:* studies with or without a comparator group were included.
- *Outcomes:* could include any eye service delivery outcome components, for example, number of service users, number of clinical assessments, number of treatments provided, and patient or health worker satisfaction.
- *Study design:* primary research reports of any study design (qualitative, quantitative and mixed methods studies). We excluded editorials, conference abstracts and posters, systematic reviews and grey literature.
- *Other:* there was no time limit on publication dates and no language limitations.

### Information sources

On 25 January 2019 an information specialist searched MEDLINE, Embase and Global Health, using the strategy published as a supplementary file with our protocol.^23^ This search was updated on 2 July 2020. All databases were searched from their inception without language limits. We examined the reference lists of all included articles to identify further potentially relevant reports of studies. We also searched the reference lists of systematic reviews that were identified during the searches.

### Selection of sources of evidence

Two reviewers (two of HB, JR, JB, LH or AB) independently screened the titles and abstracts of identified reports to exclude publications that did not meet the inclusion criteria. Full-text articles were retrieved for review if the citation seemed potentially relevant. Two of these reviewers independently assessed the full text of each report against the eligibility criteria. Any discrepancies between the reviewers were resolved by discussion, and a third reviewer was consulted when necessary.

### Data charting process

Two custom data charting forms were developed in Excel: one for studies describing implemented service delivery models in which the model is currently or has been previously applied to a population (eg, an existing spectacle supply programme for Indigenous peoples); and the other for non-implemented model reports which describe wider components of service delivery and access that have not been executed within a population (eg, a discussion of methods to deliver culturally sensitive eye care). Each form was first piloted on five studies by each of HB, JR, JB, LH and AB, and required amendments were agreed by consensus. Due to the broad scope of the studies included, data charting was an iterative process throughout the review process, with the data charting forms amended as required. Each included study was charted independently by two reviewers. Any discrepancies between the reviewers were resolved by discussion, and a third reviewer was consulted if necessary.

### Data items

For all reports we collected the following data items:

- Publication characteristics: author, title, year of publication, country in which the model was applied and type of model described (*implemented* service delivery model or *non-implemented*).
- Context: Indigenous population targeted, eye conditions targeted and clinical service provided.
- Summary of service delivery model described.

For studies describing implemented service delivery models, the following additional data items were extracted:

- Characteristics of service delivery model:
  - Indigenous engagement and cultural sensitivity (eg, whether Indigenous peoples were included in service design and implementation, methods to improve cultural sensitivity of services).
  - Service delivery inputs identified in the model (eg, human resources, medicines, surgeries, spectacles, facilities, ophthalmic equipment, health information systems).
  - Access dimensions from the Levesque model^24^ that were addressed by the model.
- Service delivery outcomes of the model if stated (eg, number of consultations, number of spectacles dispensed, number of surgeries performed, patient satisfaction).
- If the model was evaluated, and summary of the main points from the evaluation.

### Synthesis of results

The quantitative data were summarised using descriptive statistical methods (eg, measures of frequency). Qualitative data were analysed using thematic analysis. For data items on Indigenous engagement and cultural sensitivity, access dimensions and evaluation findings, data from the two data collectors were collated and read through several times for a process of familiarisation and reflection. A coding system was then developed using an iterative process of code development. The codes were then grouped into themes from which key intervention characteristics were identified. For data items on access, key themes were mapped onto the Levesque model of access dimensions.^24^

## Results

### Selection of sources of evidence

**Figure 1:**
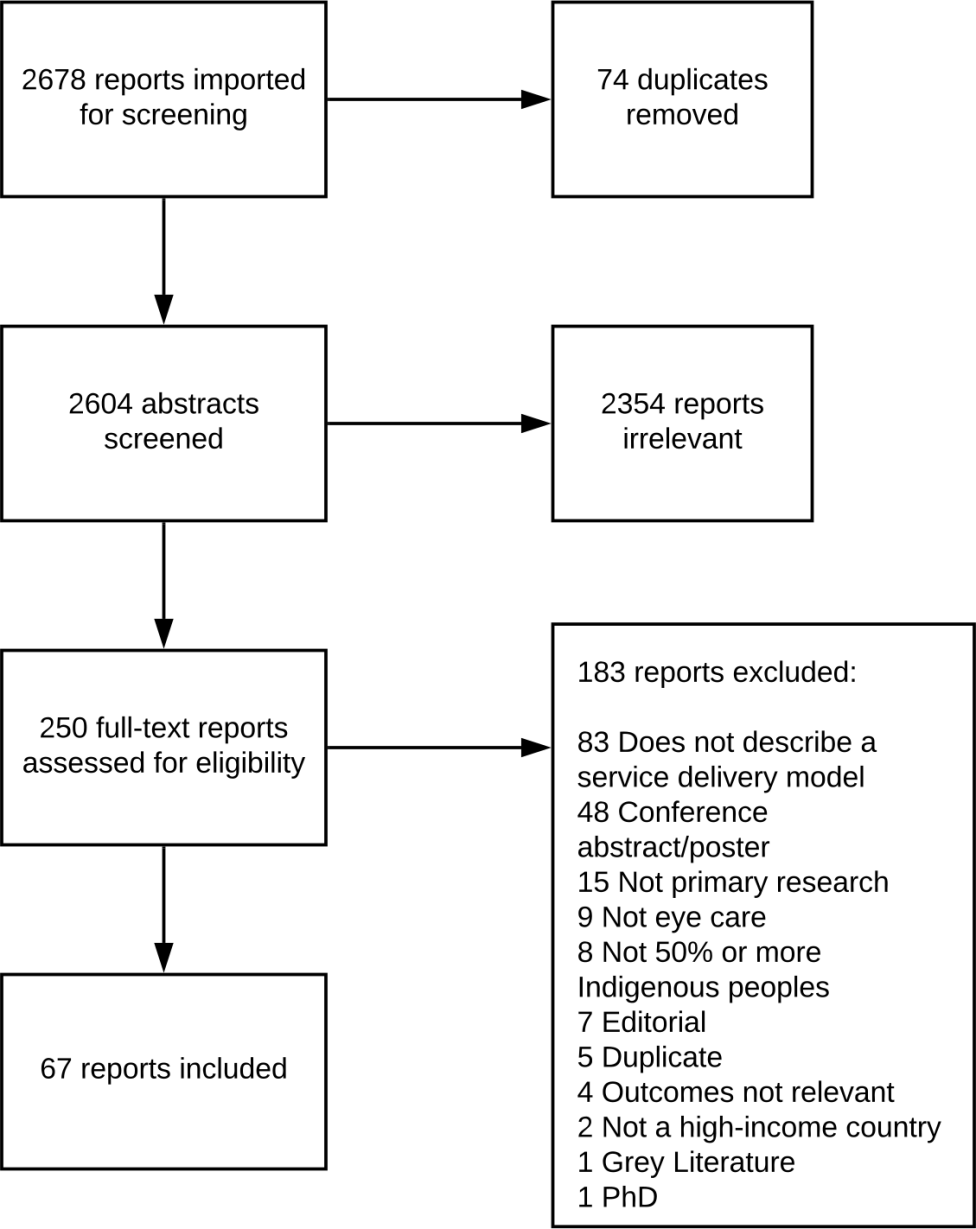
PRISMA flow diagram. PRISMA, Preferred Reporting Items for Systematic Reviews and Meta-Analyses.

We screened 2604 titles and abstracts, of which 250 full-text articles were subsequently reviewed and 67 reports ultimately included. The 67 reports represented 67 separate studies and 63 distinct models as some models were described by more than one paper (figure 1).

### Characteristics of sources of evidence

Of the 67 included studies, 50 described an implemented service delivery model (hereafter referred to as *implemented models*) that is in use, or has been in use, within a population. From these 50 studies, 46 distinct service delivery models were described. A further 17 reports discussed components of service delivery models to increase access to eye care for Indigenous populations without describing an implemented model (hereafter referred to as *non-implemented models*). Most studies focused on Indigenous peoples in Australia (n=45, 67%), followed by the USA (n=11, 16%), Canada (n=7, 10%), New Zealand (n=2, 3%), Taiwan (n=1, 2%) and Greenland (n=1, 2%); two-thirds were published after 2010 (n=45, 67%) (table 1).

**Table 1:** Characteristics of included studies

	Implemented models n=50 (%)*	Non-implemented models n=17 (%)	All reports N=67 (%)
Country which the model applies to			
Australia	31 (62)	14 (82)	45 (67)
USA	9 (18)	2 (12)	11 (16)
Canada	6 (12)	1 (6)	7 (10)
New Zealand	2 (4)	0 (0)	2 (3)
Greenland	1 (2)	0 (0)	1 (2)
Taiwan	1 (2)	0 (0)	1 (2)
Year of publication			
Pre-1980	2 (4)	0 (0)	2 (3)
1980–1989	1 (2)	0 (0)	1 (2)
1990–1999	4 (8)	2 (12)	6 (9)
2000–2009	12 (24)	1 (6)	13 (19)
2010–2020	31 (62)	14 (82)	45 (67)
Eye disease targeted			
Diabetic retinopathy	26 (52)	4 (24)	30 (45)
All eye care	8 (16)	8 (47)	16 (24)
Trachoma	9 (18)	2 (12)	11 (16)
Cataract	3 (6)	1 (5)	4 (6)
Refractive error	2 (4)	2 (12)	4 (6)
Glaucoma	1 (2)	0 (0)	1 (1.5)
Low vision	1 (2)	0 (0)	1 (1.5)
Main clinical service in the model			
Screening	26 (52)	3 (18)	29 (43)
Non-specific/general	6 (12)	8 (47)	14 (21)
Surgery (excluding trichiasis surgery)	4 (2)	1 (6)	5 (7)
Optometry	2 (4)	2 (12)	4 (6)
Health promotion/training	2 (4)	1 (6)	3 (4)
Rehabilitation	1 (2)	0 (0)	1 (1.5)
SAFE for trachoma†			
►Surgery	4 (2)	2 (12)	6 (9)
►Antibiotics	4 (2)	2 (12)	6 (9)
►Facial cleanliness	6 (12)	2 (12)	8 (12)
►Environmental change	3 (6)	2 (12)	5 (7)

*Percentage of the total number of studies for each of the three groups.

†Some trachoma reports describe more than one SAFE component.

The main disease focus of the studies was diabetic retinopathy (n=30, 45%), followed by ‘all eye care’ (n=16, 24%) and trachoma (n=11, 16%). Few studies discussed interventions to improve access for patients with cataract, refractive error or glaucoma. The most common clinical services discussed in the literature were screening (n=29, 43%), ‘general eye care’ (n=14, 21%) and surgical services (non-trichiasis) (n=5, 7%). Interventions to improve access to optometry, rehabilitation services and health promotion services for Indigenous peoples were less commonly described in the literature (table 1).

### Synthesis of results

#### Implemented service delivery models

##### Characteristics of implemented service delivery models

The 46 implemented service delivery models from 50 studies described a range of models to provide eye care to Indigenous peoples. These are summarised in table 2. Almost two-thirds were implemented in Australia (n=29/46, 63%) and almost half (n=21/46, 46%) described a teleophthalmology programme, most of which (n=19/21, 90%) were screening programmes for diabetic retinopathy. Most of these (n=12/21, 57%) described integration of teleophthalmology for diabetic eye screening into existing diabetes care in community primary care or Indigenous healthcare clinics in Australia.^27–41^ A further six models described mobile teleophthalmology services visiting Indigenous communities in Australia,^29 42^ Canada^43 44^ and New Zealand.^45 46^ One model described both these approaches and is therefore listed twice.^29^ In three of these teleophthalmology models Indigenous health workers in Australia were trained to take fundus images.^35 36 42^ Two further models from Australia focused on technical aspects of the screening programme, such as addition of optical coherence tomography imaging^47^ and the use of direct ophthalmoscopy compared with fundus cameras.^48^ A further model described mobile vision screening for children in Australia (aged 0–16 years) integrated within an existing ear screening programme visiting schools.^49^ Lastly, one model described the use of teleophthalmology to assist in preoperative assessments to reduce the waiting times for cataract surgery.^50^

**Table 2:** Key components of the 46 implemented service delivery models (50 studies in total; 3 models are described by more than one study)

Country, eye condition and eye care service	First author, year	Service delivery input	Service delivery model description	Service delivery outputs/outcomes reported	Author summary of evaluation of service delivery model
**Teleophthalmology**					
Australia DR, *screening*	Barry, 2006^29^	HR clinical. HR managerial. Equipment. HIS.	Teleophthalmology screening programmes within remote Indigenous communities in Western Australia.	Photographic quality, prevalence of DR, number undergoing annual screening and numbers referred	Increased number of people undergoing regular DR screening in Western Australia.
Australia DR, *screening*	Brazionis, 2018^30^	HR clinical. Equipment.	Teleophthalmology service using non-ophthalmic retinal imagers with remote ophthalmologist in grading images.	–	NR.
Australia DR, *screening*	Kanagasingam, 2015^37^	HR clinical. Equipment. Facilities. HIS.	Teleophthalmology model ‘*Remote-I*’ connected ophthalmologists in urban areas to patients and primary care workers in remote locations, including secure image sharing.	Patient satisfaction, numbers screened and health professional satisfaction	Good patient and health professional satisfaction and an increased number of patients screened.
Australia DR, *screening*	Karagiannis, 1996^42^	HR clinical. Equipment.	Pilot study to assess whether Indigenous health workers could be trained to take fundus images for retinal screening to reduce cost of DR screening in remote areas rarely visited by an ophthalmologist.	–	NR.
Australia DR, *screening*	McConnell, 1993^48^	HR clinical. Equipment.	Indigenous healthcare workers taking fundus photos on health visits and sending photos to ophthalmology consultant.	–	NR.
Australia DR, s*creening*	Moynihan, 2017^36^	HR clinical. HR managerial. Equipment. Facilities.	Retinal photography and visual acuity measured by Indigenous health workers and nurses and sent via cloud-based eHealth records systems to Perth-based ophthalmologist. A Kimberley diabetic eye health coordinator was established to provide high-level support.	Change in coverage provided by the screening programme over time and number of centres involved in screening programme and number of diabetic eye examinations carried out annually	With the addition of an eye health coordinator, coverage for Indigenous patients increased and number of sites for screening increased.
Australia DR, *screening*	Murray, 2005^35^	HR clinical. Equipment.	Indigenous health workers trained to operate non-mydriatic retinal cameras to screen for DR in primary care clinics. Photographs were reported by remote ophthalmologists. Regular training and feedback were provided to the technicians.	Number of screening episodes	Evaluated the activities and outcomes of the last 5 years of this 10-year programme. Showed the number of Indigenous population screened increased after starting programme.
Australia DR, *screening*	O’Halloran, 2018^47^	HR clinical. Equipment.	Assessed the addition of an OCT alongside fundus camera to improve detection of DR.	Referral rates from screening to eye health professional	Addition of OCT did not change number of referrals.
Australia DR, *screening (same model described)*	Spurling, 2010^38^	HR clinical. Equipment.	Clinic-based retinal photography introduced to urban Indigenous health service primary care.	Access to appropriate screening and follow-up, acceptability and feasibility of clinic-based retinal photograph	Number screened, referred and followed up increased. Participants were positive about the screening (convenient and in a comfortable/safe environment).
	Villalba, 2019^31^	HR clinical. HR managerial. Facilities. HIS. Equipment.		Number attending DR screening	Implementation of a retinal camera at the CoEinitially increased DR screening rates. From 2012 to 2016, the annual DR screening number was consistent, but the number of CoE clients living with diabetes increased substantially.
Australia All eye care, *screening*	Elliott, 2010^49^	HR clinical. Equipment. Facilities. HIS.	Integration of a mobile telehealth hearing and vision screening service for children with existing community-based health services. Screening carried out in a mobile van by an Indigenous health worker. Children who failed screening were referred to local health services.	Community acceptance, number of schools visited, number of children screened and number of referrals made to optometry	Combining telehealth ear screening with eye screening using Indigenous health workers is likely to be feasible.
Australia Cataract, *surgery*	McGlacken-Byrne, 2019^50^	HR clinical. Surgeries. HIS.	Teleophthalmology was used by optometrists for preoperative consultations during outreach visits, allowing patients to be booked for cataract surgery without the need for outpatient preoperative assessment.	Cataract surgery waiting times	Almost one-fifth of cases (19.1%) had surgery booked via telehealth, resulting in shorter waiting times between referral and cataract surgery.
Canada DR, *screening*	Arora, 2013^34^	HR clinical. Equipment.	Teleophthalmology programme in Alberta, Canada incorporating culturally sensitive health-related activities and rituals as a component of DR screening. Nurses held clinics once a month in remote communities. Digital fundus photographs are sent to ophthalmologists remotely, who reviewed images and suggested management.	Clinic attendance rate and patient satisfaction	Teleophthalmology is more effective than the traditional hospital-based approaches at resolving social and cultural barriers, thereby facilitating greater access to care for remote Indigenous peoples.
Canada DR, *screening*	Kim, 2015^43^	HR clinical. HR managerial. Equipment. Facilities.	Teleophthalmology screening and follow-up for at-risk patients with diabetes. Patients phoned and invited to attend screening clinics in their communities. Clinic arrived in a truck and set up at existing healthcare centres within remote communities.	Number screened, user satisfaction, system quality, service quality and costs	Increased number of people screened, user satisfaction with teleophthalmology was high, indirect and direct cost savings, and able to diagnose other ocular conditions.
Canada DR, *screening*	Jin, 2004^44^	HR clinical. Facilities. Clinical episodes.	The British Columbia First Nations Mobile Diabetes Telemedicine Clinic visits Indigenous communities once per year. A diabetic nurse educator and eye care technician carry out screening alongside other diabetes care and diabetes education.	Number and location of clinic sites, number of clinic days of operation, number of clients examined, client satisfaction, and cost-effectiveness	The mobile diabetes clinic programme provides a relevant and needed service that is effective, with high satisfaction from patients and delivered at less cost than the existing alternative.
Canada DR, *screening*	Spurr, 2018^28^	HR clinical. Equipment. Facilities.	A pilot study to see whether community-based, nurse-led screening for DR was as accurate as an assessment by an ophthalmologist.	–	Showed agreement between nurse-led and ophthalmology screening, however small pilot study not assessed sustainable implementation yet.
New Zealand DR, *screening*	Jagadish, 2017^46^	Facilities.	DR screening using a mobile retinal screening van in a range of settings. Patients referred to specialist eye clinic after grading.	–	NR.
New Zealand DR, *screening*	Reda, 2003^45^	HR clinical. Equipment. Facilities.	Mobile teleophthalmology screening clinic in Waikato region.	Number of patients screened, number referred to eye clinic and failure to attend rate	Reports good coverage in the area and acceptable image quality.
USA DR, *screening*	Bowyer, 1997^27^	HR clinical. HR managerial. Medicines.	Describes the experiences of the White Earth Indian health centre in establishing a pilot project for the prevention of ocular complications of diabetes. 13 quality improvement steps were designed to improve diabetic eye care within the primary care clinic.	Number attending annual eye examination	Increase in number of people having annual diabetic eye examination.
USA DR, *screening (same model described)*	Carroll, 2011^40^	Equipment. HIS.	The Indian Health Service-Joslin Vision Network Teleophthalmology Program was established in 2000 for remote diagnosis and management of DR. Non-mydriatic fundus photographs are taken by a technician as a routine component of diabetes care. Retinal images are sent to a central server on the Indian health service network for review. Currently has 99 clinical implementations in 23 states.	Number of screening episodes	States general increase in numbers screened and geographical coverage. Some sites still demonstrate a low utilisation rate, possibly due to not being included in standard diabetic care.
	Bursell, 2018^39^	HR clinical. Facilities.		–	NR.
	Fonda, 2020^33^	HR clinical. Facilities. HIS. Equipment.		Clinical volume, geographical adoption of programme, DR surveillance and treatment rate, cost-effectiveness, and operation efficiency	Annual DR screening rate has increased within HIS sites.
USA DR, *screening*	Mansberger, 2013^41^	HR clinical. Facilities. HIS.	Non-mydriatic fundus photographs taken during primary care clinic for routine diabetes care by trained technicians in a vacant room within the clinic. Images were then sent remotely for grading.	Number attending screening	Teleophthalmology with non-mydriatic camera increased uptake of screening compared with traditional clinic-based surveillance.
Greenland DR, *screening*	Pedersen, 2019^32^	HR managerial. HIS. Ophthalmic equipment.	Non-mydriatic wide-field digital fundus photographs are taken by nurses working in diabetic clinics and images then sent remotely to ophthalmologists in Denmark. Cameras were installed in nine towns and local diabetes staff were trained in the procedure.	Number attending screening	Increase in number of patients undergoing annual DR screening since programme initiated in 2008.
**Integration of services into existing Indigenous primary health**					
Australia DR, *screening*	Bailie, 2007^54^	HR clinical. HIS.	Two cycles of a quality improvement intervention in 12 Indigenous community health centres with a focus on understanding system-related factors which hinder or facilitate improvements in outcomes of care in Indigenous peoples with diabetes.	Change in quality of diabetic care and change in health centre system development	Increase of biennial diabetic eye screening by an ophthalmologist from 34% to 54%.
Australia Refractive error, *optometry*	Layland, 2004^53^	HR clinical. HR managerial. Facilities. Spectacles supplied. Surgeries.	Local, culturally appropriate, fully equipped remote eye clinics, some staffed by optometrists and others visited by optometrists, with clear referral and funding pathways and integration with more remote communities. The clinics provide eye care, spectacle dispensing and eye health education.	Number of spectacles supplied to Indigenous population, number of ophthalmic consultations and number of eye care clinics in remote areas	Increased use of government spectacle scheme by Indigenous people, good relationships with community with continuity of care and good feedback from Indigenous communities.
Australia Refractive error, *optometry*	Napper, 2015^78^	HR managerial. Spectacles supplies.	New low-cost spectacle scheme (the Victorian Aboriginal Spectacle Subsidy Scheme) and expansion of service access sites in urban and regional Victoria aimed at improving access to and uptake of eye care for Indigenous Australians.	Number of patient consultations, number of spectacles dispensed and number of patient referrals	Increased access to spectacles, improved management of other eye conditions, improved referrals for systemic conditions and increased participation of the Aboriginal health service in eye care services.
Australia Cataract, *surgery*	Penrose, 2018^57^	HR clinical. Medicines.	The Institute for Urban Indigenous Health (IUIH) introduced ‘wrap around’ culturally appropriate care that extends to tertiary services. Collaborations to redesign services took place between clinicians, community members and an Indigenous health institute (IUIH).	Number of referred patients undergoing cataract surgery in the 7-month period following service redesign, 4-week postoperative check attendance rates and visual acuity outcomes following surgery	Integrating the cataract surgical pathway within the primary healthcare service and collaborating with external organisations improved coordination and increased the cataract surgery completion rate for Indigenous Australians with high-quality visual outcomes.
Australia DR, *screening*	McDermott, 2001^55^	HR clinical. Facilities. HIS.	A diabetes recall system was established at 21 primary healthcare sites. Indigenous healthcare workers were trained to manage a recall system and provide diabetes checks and referrals.	Number undergoing annual DR screening	Patients with diabetes at intervention sites had more frequent, regular and structured contact with the primary healthcare service. More patients underwent an annual eye check in the intervention group.
Australia All eye care, *general*	Yashadhana, 2020^59^	HR clinical. Surgeries. Spectacles. HIS.	Strategies to improve access to eye care and its integration with regional health systems were implemented following a situational analysis. Activities included training of primary eye care staff, documentation of referral processes, updating e‐record templates to include primary eye care examinations and increasing visiting optometry and ophthalmology services.	Number of eye care attendances, spectacles dispensed, dilated fundus examinations, referrals and cataract surgeries completed	There were significant increases in the rate and frequency of optometry examinations, recalls and referrals and spectacles prescribed and annual dilated ocular fundus examinations.
Australia All eye care, *general*	Jatkar, 2017^58^	Surgeries. Spectacles. Equipment.	Overview of the Grampians Region Aboriginal Eye Health Advisory Group eye care programme. Gap analyses were carried out which led to purchasing ophthalmic equipment, providing eye care training to staff, developing DR health promotion resources, implementing strategies to reduce waiting times for cataract surgeries and making spectacles more affordable.	Visits to optometry, number receiving annual DR screening, waiting times for cataract surgery and cataract surgical rates	Increased visits to optometry, number attending annual DR screening, number receiving cataract surgery and number receiving subsidised spectacles. Decrease in waiting time for cataract surgery.
USA Refractive error, *optometry*	Caplan, 1978^51^	HR clinical. Spectacles supplied.	Eye clinics and children’s visual screening integrated within the Indian health service.	Number of optometrists working *within Indian health service*	Number of optometrists working within the *Indian health service* increased during the 1970s, but no wider evaluation of access to services.
Canada DR, *screening*	Hayward, 2020^56^	HR clinical. HR managerial.	The programme partnered with a team of local healthcare providers and community members to develop and evaluate a community-driven, culturally relevant primary healthcare model using a QI process. They aimed to improve diabetes care access to prevention services in the community, including DR screening.	Number attending DR screening	Most aspects of general diabetes care were improved, however the number receiving DR screening decreased.
**Outreach services**					
Taiwan All eye care, *general*	Chen, 2015^60^	HR clinical. HR managerial. Equipment. Facilities. Spectacles supplied.	Mobile van providing eye screening in Indigenous communities, with on-board dispensing of spectacles.	Number of primary eye care services provided, number of spectacles dispensed, number of eye health education programmes and courses given	This eye care model is feasible and cost-effective.
Australia All eye care, *general*	Gruen, 2006^61^	HR clinical. Clinical episodes.	Visiting ophthalmic specialists to remote communities. Patients with non-emergency surgical problems were referred from primary care to these outreach clinics.	Number of elective referrals, number of opportunistic attendances, proportion of electively referred problems seen by a specialist within 12 months and timely completion of referrals	Specialist outreach visits to remote Indigenous communities improved access to specialist consultations and procedures without increasing elective referrals or demand for hospital inpatient services.
Australia All eye care, *general*	Maher, 2012^63^	HR clinical. HIS. Spectacles supplied. Surgeries. Clinical episodes.	Overview of ophthalmic services available in New South Wales providing several examples of service delivery models.	Cataract surgical rates, availability of eye health services, access to services, coordination of services, and monitoring and evaluation of services	Identified areas for improvement: a lack of cultural competency, limited coordination, and incomplete monitoring and evaluation.
USA Glaucoma, *surgery*	Robin, 1986^64^	Equipment. Medicines.	A field trial of the use of a portable Nd-YAG laser for peripheral iridotomies in rural villages to prevent pupillary block glaucoma in at-risk patients.	–	NR.
Australia DR, *general*	Turner, 2011^62^	HR managerial. Medicines.	Describes models for service integration between ophthalmology and optometry when conducting outreach eye services.	Surgery and clinic consultation rates, waiting times and costs per attendance	Better integration of optometry and ophthalmology services increases surgical uptake rate.
**Training Indigenous health workers**					
Australia All eye care, *education, training*	King, 2003^66^	HR managerial.	Developed an eye course for health workers from two Aboriginal communities and produced health promotional materials to educate clients in the Aboriginal community about eye health issues.	–	NR.
USA Low vision, *rehabilitation*	Orr, 1993^65^	HR clinical. Facilities.	Training model to teach community outreach workers to train elderly blind and vision-impaired American Indians independent living skills.	–	NR.
**Eye health promotion**					
Canada DR, *health promotion*	Umaefulam, 2020^67^	HIS.	This study explored the use of mobile health (mHealth) via text messages to provide DR awareness and improve diabetic eye care behaviour. It examined the extent to which mHealth education changed Indigenous women’s DR awareness and self-reported eye care behaviour.	KAP related to DR	Improvement in KAP related to DR among participants.
**Trachoma control methods**					
Australia Trachoma, *SAFE (F)*	Atkinson, 2014^72^	HR managerial. Clinical episodes. Facilities.	The University of Melbourne partnered with Melbourne Football Club to run trachoma football hygiene clinics in Northern Territory, Australia, to raise awareness of the importance of clean faces in order to reduce the spread of trachoma. Between 2010 and 2013, 12 football clinics were held in major towns and remote communities.	Number attending the football clinics	Engagement in football clinics, number of communities involved and media coverage increased between 2010 and 2013.
Australia Trachoma, *SAFE (AFE)*	Ewald, 2003^70^	Medicines. Clinical episodes.	Implementation of the SAFE strategy in central Australia: screening children, antibiotic distribution, health promotion and environmental improvements.	Prevalence of active trachoma and adequacy of housing facilities	Change in trachoma prevalence after initiation of programme was not significant. Likely to be affected by population mobility, inadequate housing, continued crowding and low compliance with antibiotic therapy.
USA Trachoma, *SAFE (A)*	Hoshiwara, 1971^68^	HR clinical. Medicines.	Upscaling of mass antibiotic distribution using a family treatment approach to treat and prevent trachoma among Indigenous peoples in the USA.	Number receiving antibiotic treatment	Reduction in prevalence of active trachoma.
Australia Trachoma, *SAFE (FE)*	Lange, 2017^71^	HR managerial. Clinical episodes.	Development of a culturally appropriate, community-based health promotion strategy for trachoma using a variety of different methods for dissemination, for example, clinical education, community performances, football, Trachoma Story Kits, posters, television and radio advert.	KAP	Health promotion was associated with increased trachoma KAP among health, education and community support staff working with children and in remote communities.
	Baunach, 2012^77^	HR clinical.	Describes the process of developing and rolling out culturally appropriate health promotion resources for trachoma through collaboration of different stakeholders.	Qualitative outcomes of user satisfaction	Engaging and contemporary health promotion resources are vital to support health promotion in trachoma. Highlights the role of effective partnerships to create resources developed by Indigenous peoples for Indigenous peoples in remote communities.
Australia Trachoma, *SAFE (AFE)*	Lansingh, 2010^73^	HR managerial.	Study to assess the additional impact of implementing environmental changes within the SAFE strategy in controlling trachoma in two Indigenous Australian populations.	–	NR.
Australia Trachoma, *SAFE (A)*	Liu, 2016^69^	HR clinical. Medicines. Clinical episodes.	Annual screening of children for trachoma in communities designated to be at high risk of disease and treatment of those affected with the antibiotic azithromycin.	Number of screening episodes	Change in trachoma prevalence based on treatment strategy; most effective in communities implementing community-wide strategies.
Australia Trachoma, *SAFE (S)*	Mak, 2001^74^	Surgeries. Clinical episodes.	A collaborative programme was established involving the Kimberley Public Health Unit, Kimberley Aged Care Services and the visiting ophthalmology service. The aged-care population were screened for trichiasis and the aged-care services staff were educated about identification and referral procedures for patients with trichiasis.	Number of people screened for trichiasis and number referred for surgery	An effective screening programme and referral system improves access to trichiasis surgery.
Australia Trachoma, *SAFE (AFE)*	Mak, 2006^76^	HR clinical. Medicines. Clinical episodes.	Provides an overview and comparison of the different trachoma control strategies in different regions of Australia in 2004.	Number of screening episodes	Trachoma control programmes led by regional population health units working in collaboration with primary healthcare services were more likely to be consistently implemented over long periods of time.

DR, diabetic retinopathy; HIS, health information system; HR, human resources; KAP, knowledge, attitude and practice; NR, not recorded; OCT, optical coherence tomography; QI, quality improvement; SAFE, surgery, antibiotics, facial hygiene and environmental change; CoE, Centre of Excellence in Primary Care.

Beyond integration of teleophthalmology services, nine models described the integration of other eye care services into existing Indigenous primary healthcare. Three models focused on optometry services in the USA^51^ and Australia.^52 53^ Three described the integration of eye care into comprehensive diabetic services in Australia^54 55^ and Canada.^56^ One focused specifically on cataract services in Australia,^57^ and two described general eye care programmes in Australia.^58 59^

Another described model was the use of outreach services (n=5). Four papers described outreach to rural and remote locations by optometrists and/or ophthalmologists: one in Taiwan^60^ and three in Australia.^61–63^ One paper described mobile laser surgery for narrow angle glaucoma prevention in Canada.^64^

Two models described the use of training Indigenous health workers to deliver community eye care services: one trained community outreach workers to teach vision-impaired older people independent living skills in the USA^65^ and the other trained Aboriginal health workers in primary eye care in Australia.^66^ One model described the use of mobile phones (mobile health) to carry out eye health promotion regarding diabetic retinopathy with Indigenous women in Canada.^67^

Lastly, eight models focused exclusively on access to trachoma control measures in the USA (n=1)^68^ and Australia (n=7).^69–77^ Trachoma requires specific, well-defined interventions which are not widely applicable to other eye conditions: SAFE (surgery for trichiasis, mass antibiotic distribution, promotion of facial hygiene and environmental change). One model focused specifically on screening for trichiasis,^74^ two models described mass antibiotic distribution,^68 69^ and three others focused on all aspects of trachoma except trichiasis surgery.^70 73 76^ One model described a health promotion strategy to increase facial hygiene practices using clinics at football matches,^72^ and another described a multicomponent health promotion strategy for trachoma implemented in Northern Territory, Australia.^71 77^

##### Effectiveness of implemented service delivery models

###### Reported outcomes and evaluations

Of the 50 studies reporting an implemented model, 41 (82%) reported at least one outcome related to access (table 2). These studies also provided some reflection on whether the eye care service delivery model was successful, but this tended to draw on changes in service outputs, such as consultations or spectacle dispensing, without any statistical or comparative analysis. No studies evaluated the long-term impact, for example, change in the burden of vision impairment, among an Indigenous population as a result of implementing a service delivery model (table 2).

###### Indigenous engagement

Authors included an explicit statement that the model was designed to be socially and/or culturally appropriate in 20 (40%) implemented service delivery model studies. Strategies included employing regional eye health coordinators from within the Indigenous community to improve coordination between healthcare providers and the community^49 60^ and using Indigenous health workers to carry out community eye care that is culturally sensitive.^35 43^ A study in Canada demonstrated that a culturally sensitive, community-based teleophthalmology clinic for Aboriginal Canadians significantly increased attendance rates,^34^ while attendees of a community-based diabetic retinopathy screening within an Indigenous health service in metropolitan Australia reported the screening experience was more ‘culturally safe’.^38^ One example of a culturally sensitive trachoma programme was the multicomponent health promotion model implemented in Northern Territory, Australia. Several different health promotion initiatives were centred around the ‘Trachoma Story Kit’, developed as a culturally appropriate health promotion material with input from Aboriginal health services, Departments of Health and Education, non-governmental organisations, community programmes and environmental health. The implementation of the programme was advised throughout on cultural safety and acceptability by the Ngumbin Reference Group of Elders and Aboriginal health workers.^71 77^

Authors reported that Indigenous peoples were involved in the design of the eye care service in 17 of the 50 studies (34%). For example, when establishing a spectacle subsidy scheme in Victoria, Australia, the leaders of the target community were included as stakeholders, community elders were involved in the selection of spectacle frames, and an Indigenous patient pathway coordinator was selected from the community.^52^ In a teleophthalmology screening programme in Canada health providers sent letters to community leaders to assess interest in the scheme, invited community members to attend project launch meetings and organised clinic dates and times based on preferences of the community.^43^ Other studies described how Indigenous community members and/or leaders were consulted to help design eye care services. For example, in a cataract surgical service redesign programme in Australia, community members from the target Indigenous population were involved in brainstorming the service redesign.^57^ In Canada ideas for making a screening programme more culturally acceptable were gained from consulting a spiritual liaison from the Indigenous community.^34^

###### Access dimensions

**Figure 2:**
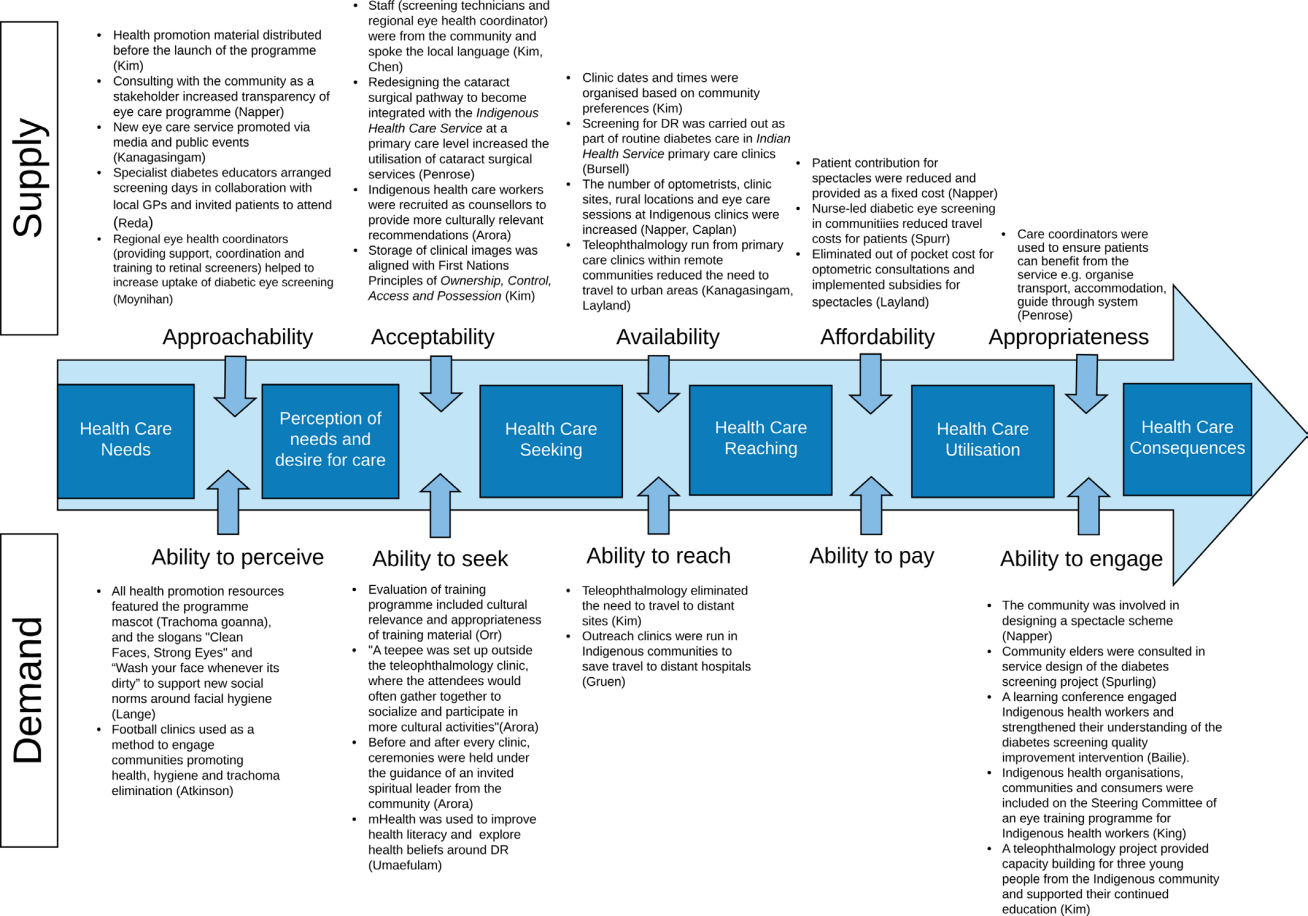
Components of identified service delivery models to improve access to eye care for Indigenous peoples, mapped against the stages of the Levesque *et al*^24^ framework (illustrative examples extracted from 50 implemented model studies). DR, diabetic retinopathy; GP, general practitioner; mHealth, mobile health.

Only 2 of the 46 implemented service delivery models described a model that addressed all stages of access outlined in the Levesque *et al*^24^ access framework; these and other models are mapped against Levesque *et al*’s framework in figure 2. The first model addressing all access stages involved teleophthalmology screening for First Nations clients with diabetes in Canada.^43^ This programme distributed health promotion material before the launch of the programme, eliminated travels costs and time for the client by bringing the clinic to the community, provided screening free of charge, and aimed to engage the community through supporting young people from the targeted community to gain work experience within the scheme. Second, a low-cost spectacle scheme implemented in Victoria, Australia increased transparency through use of members of Indigenous community as stakeholders. They improved accessibility by increasing the number of service sites, clinical sessions, optometrists and expanded to more rural locations. The spectacles were provided at a reduced fixed cost, services were provided within the existing culturally appropriate *Aboriginal health service* facilities, and community engagement was undertaken to plan and implement the scheme.^78^

Across all studies, ‘availability/ability to reach’ was the access stage most often addressed, with 44 studies reporting some aspect of increasing availability of eye services for Indigenous peoples. This commonly involved increasing the number of clinical sites,^51 52^ providing services in more remote/rural areas,^43 61^ expanding human resources^51 52^ or providing more flexible clinic operating times.^43^ Other access stages were described by far fewer studies, for example, ‘appropriateness/ability to engage’ (n=17). Examples exclusively focused on improving the ability of the community to engage in the eye care service design and implementation.^38 52 54 66^ ‘Acceptability/ability to seek’ was addressed in 18 studies; examples included employing a community eye health coordinator,^36 49 57 60^ recruiting health workers from the targeted Indigenous community^34 43 60^ and liaising with existing Indigenous healthcare systems.^39 45 57 61^ In terms of ‘approachability/ability to perceive’ (n=18), the literature provides some positive examples of increasing community awareness of eye care services to improve approachability,^36 37 43 45 52^ but there were fewer examples of attempts to improve a community’s ‘ability to perceive’.^71 72^ ‘Affordability/ability’ to pay was rarely reported (n=7). There were no examples of improving ‘ability to pay’, but some delivery models described fixed, low-cost or free eye care services.^52 53^ Across all five access dimensions, models on the supply side were more commonly described than the demand side.

#### Non-implemented models

An additional 17 reports were identified that discussed wider aspects of service delivery and access to eye care for Indigenous peoples in high-income countries, but did not specifically describe or evaluate an implemented service delivery model. Key strategies for improving access to eye care were drawn from these reports and are summarised in table 3. These strategies have been matched with the five service delivery models described in the ‘Implemented service delivery models’ section, and an additional category of broader themes has also been included.

**Table 3:** Strategies for improving access to eye care for Indigenous peoples proposed in reports of non-implemented models (n=17)

Service delivery model	Proposed strategies to improve access
Teleophthalmology screening	Ensure culturally appropriate ‘patient-provider’ communication regarding diabetic retinopathy to reduce non-adherence to screening programmes.^86^
Eye care integrated within primary care and Indigenous healthcare	Train primary care staff to enable primary care to support eye care delivery.^62 81 87–90^ Design tertiary ophthalmic services to incorporate needs of Indigenous peoples to improve uptake.^72 88^ Ensure primary healthcare staff have knowledge of a well-defined patient pathway from community identification of eye care need to referral, treatment and monitoring.^91^
Outreach ophthalmology and optometry	Improve coordination between Indigenous healthcare system and eye care services.^62 87–90 92–94^
Education, training and health promotion	Provide culturally appropriate eye health knowledge training for healthcare workers and their communities.^70 90 95^
Trachoma control measures	Optimise the intensity and strategy of comprehensive interventions in Australian Aboriginal communities with endemic trachoma.^70 92 96–98^
Broader service delivery components	Use a health systems approach when designing eye care services.^90^ Calculate estimates of cost, cost-effectiveness and models of funding eye care.^88 90 99 100^ Deliver culturally appropriate eye care services to reduce barriers to patient access.^11 88 89 101^ Distribute eye care professionals and funding equitably.^13 62 89^ Plan sustainable eye care services.^101^ Ensure monitoring and evaluation to collect, analyse and report local, regional and national data on eye care services, quality and satisfaction.^89 90^

## Discussion

### Summary of evidence

#### What were the numbers and characteristics of published reports describing service delivery models to improve access to eye care for Indigenous populations in high-income countries?

Most studies identified in this review described eye care delivery models to improve access to eye care for Indigenous peoples in Australia and focused on the delivery of diabetic eye care services or trachoma elimination strategies. There was very little literature from other high-income countries with Indigenous peoples such as New Zealand, Canada, USA, Taiwan and Greenland, and none from countries such as Singapore, Uruguay and Chile.^9^ This lack of evidence is despite documented inequality in the prevalence of ocular conditions among Indigenous populations in many of these countries. In their recent systematic review, Foreman *et al*^9^ identified the leading causes of vision impairment (visual acuity worse than 6/18) among Indigenous adults worldwide were uncorrected refractive error (responsible for an estimated 54.0%–65.1% of vision impairment) and cataract (20.1%–29.3%). The relative magnitude of these two conditions is not reflected in the studies we identified; only four studies specifically focused on the delivery of optometry and/or refraction services to Indigenous peoples,^51 52 79 80^ while a further four focused on cataract services^50 57 61 81^ (table 1).

#### What methods and interventions are used by the identified service delivery models to improve access to eye care for Indigenous populations?

There was consistency among the studies in the service delivery models described. The most commonly described model was teleophthalmology, specifically the integration of teleophthalmology screening into existing primary care and/or Indigenous healthcare services. This model is in line with recommendations outlined in Australia’s *Roadmap to Close the Gap for Vision*^82^ and aims to improve the identification and referral of eye care needs from the primary care setting. The use of outreach services to Indigenous peoples was described less frequently in the literature.

Fewer than half of the studies reporting implemented models discussed methods to make eye care service delivery socially and culturally acceptable to the Indigenous peoples being targeted, and even fewer reported involving Indigenous peoples in the design and implementation of services. This contrasts with the message repeatedly drawn from the non-implemented model studies that services need to be culturally appropriate in order to overcome the documented barriers to access to eye care among Indigenous peoples. Clearly there is a gap between what is discussed as a theoretical ‘gold standard’ approach to providing eye care to Indigenous peoples and what is carried out in practice. Some studies in this review provided examples of how this can be achieved, such as the Canadian teleophthalmology screening service that placed a teepee outside the clinic for patients to participate in cultural activities such as bracelet making and sharing of food, and to enable patients to discuss their physical, mental, spiritual and emotional health as part of their eye healthcare.^34^

Most of the models we identified in the literature (eg, teleophthalmology, integration into Indigenous primary health, outreach, training Indigenous health workers) aimed to keep eye care within Indigenous communities. Despite this, very few studies referred to cultural responsiveness, cultural safety or engagement. If health service interventions are brought into communities, but are not engaged with communities, eye health outcomes are more likely to remain the same. Service delivery models need to adopt frameworks that place cultural responsiveness at their core. For example, a framework to provide culturally responsive service delivery to Indigenous peoples has been developed by the Indigenous Allied Health Australia.^83^ The cultural responsiveness framework provides practical strategies to enable health services to provide culturally safe and responsive services that meet the needs of Indigenous peoples, with continued dialogue and engagement with Indigenous peoples placed at the core of the framework.

The literature identified tended to describe supply-side, rather than demand-side strategies, including methods to improve availability such as scheduling clinical services more frequently and having services located in isolated communities. Few studies described ways in which the arguably more challenging components of service delivery (appropriateness/ability to engage, acceptability/ability to seek and approachability/ability to perceive) could be improved. These demand-side access dimensions (ability to engage, seek and perceive) have been discussed in the wider literature as important areas to target to improve access to eye care. A recent qualitative study of determinants of eye health among Indigenous Australians with diabetes found that trust, culture and communication were three key areas in need of improvement in order to improve patient access to eye services and eye health outcomes.^11^ In particular, this study found that a lack of Indigenous language interpreters, lack of cultural literacy for non-indigenous clinicians and distrust of clinicians and health services contribute to reduced access to and uptake of eye care services. One proposal is the introduction of Indigenous liaison officers to increase cultural safety and trust in hospital settings and provide advocacy and non-clinical support. These non-clinical roles could supplement the role of eye health coordinators which have been commonly mentioned in the literature in this review. In addition, cultural responsiveness training for non-Indigenous clinical staff is a further tool that can be used to enable culturally safe access to eye care services.

Very few studies discussed affordability, despite cost often being stated as a barrier to accessing eye care.^84^ The preponderance of reports from Australia may explain this, given that government funding is available for eye care examinations by ophthalmologists and optometrists. The review demonstrated a lack of rigorous evaluation of service delivery with regard to access dimension outcomes. No study provided a comprehensive evaluation of outcomes, such as the change in the burden of vision impairment within the population where the eye care service was delivered. Instead, many studies reported on project outputs, such as change in patient attendance numbers.

#### What are the current gaps in the literature and what lessons can be learnt regarding models that have been successful in improving access?

Promisingly, most studies were published after 2010, suggesting that the published literature on this topic is increasing over time. However, while the volume is increasing, the scope remains narrow. The geographical spread needs to increase, in particular with more research outside Australia, and the inclusion of other high-income countries with marginalised Indigenous peoples, for which no literature was identified in this review. Many of the service delivery components, particularly those outlined in the non-implemented model reports, are applicable to most nations and should not be limited to one country or region. However, it is important that implementation research is conducted in the setting within which the service is needed and ensures local community engagement and ownership.^6^

The review also identified a limited clinical scope. We recommend further research beyond the current focus on diabetic retinopathy screening. This service requires specialist photography equipment, health information systems and technologies which are not always applicable to other aspects of eye care, and therefore findings from these studies lack generalisability to other eye care services. The review revealed a major gap in reporting the short-term and long-term clinical outcomes of eye care services developed for Indigenous peoples in high-income countries. Outputs (numbers attending, satisfaction surveys) were more commonly reported in the literature. Without more comprehensive evaluations of services, the success of delivery models remains largely unknown.

Lastly, the methodological approaches used could be strengthened to provide more robust evidence for interventions that are effective. Although this review, as a scoping review, did not formally assess the quality of included studies, we were able to identify a lack of fully evaluated long-term implementation studies to robustly assess a service delivery model for eye care in this setting. Although service delivery outcomes were collected for most studies, few studies evaluated the model using all five access dimensions and none was able to show the long-term impact of the intervention.

### Limitations

There are some differences between our published protocol^23^ and this scoping review. First, there were a larger number of studies identified in the literature search than we had predicted. We therefore decided not to include grey literature as we felt the published literature alone would answer our research questions and we were limited by time and resources to additionally search all grey literature on this topic. We are aware that this scoping review includes only models that have been published in the peer-reviewed literature and therefore may not reflect all service delivery models in use. Second, in the protocol we stated we would collect information on the ‘enabling health system functions’ described in the model. As we started the iterative process for data charting it became clear that these data items were rare within the reports and better captured by other items we collected and analysed, such as the description of the intervention. Third, the literature was very heterogeneous, and in attempting to keep the scope very broad we had to analyse reports in different ways, as some did not describe implemented service delivery models. Although we could not extract from these reports the data items we had proposed to extract in our protocol, we decided to retain ‘non-implemented model reports’ as they provided information on wide-reaching themes around the subject, which have helped to deepen our understanding. As we were completing our review, the CONSIDER (Consolidated criteria for strengthening reporting of health research involving indigenous peoples) statement was published providing guidelines for strengthening the reporting of health research involving Indigenous peoples.^85^ Our team includes Indigenous researchers and people who have worked in Indigenous primary healthcare and Indigenous eye care. However, we recognise that our review falls short of the CONSIDER statement in several areas, and in particular could have been further strengthened had we engaged Indigenous peoples with vision impairment. Lastly, as the majority of Indigenous peoples live in low-income and middle-income countries, by focusing only on high-income countries in this review it is likely we will have missed an important proportion of eye care services among Indigenous peoples living elsewhere. This will provide an important next research priority.

## Conclusions

This scoping review identified a narrow geographical and clinical focus within the published literature on service delivery models to improve access to eye care for Indigenous peoples within high-income countries. The geographical locations, eye diseases targeted and eye care services delivered do not reflect the epidemiology of eye disease among Indigenous peoples in high-income countries. In 2015, the UNPFII called for culturally, linguistically and geographically appropriate models of care for Indigenous peoples, as well as participation by Indigenous peoples in the design and implementation of health policies and programmes.^6^ Disappointingly this review has found few examples of this approach in published studies. There are isolated examples of improvements in access to eye care when services are developed in partnership with Indigenous peoples. However, to realise the SDGs and *leave no one behind*, much more must be done to ensure Indigenous peoples can access eye care.
